# Utilization of Voluntary Counseling and Testing Experience among Mizan-Tepi University Students in Southwestern Ethiopia

**DOI:** 10.1155/2022/7911385

**Published:** 2022-07-18

**Authors:** Lema Abate Adulo, Sali Suleman Hassen, Admasu Markos Kontuab

**Affiliations:** Department of Statistics, College of Natural and Computational Sciences, Mizan-Tepi University, Tepi, Ethiopia

## Abstract

**Background:**

Voluntary counseling and testing (VCT) is the primary gateway to HIV prevention, caution, and handling, where people learn whether they are infected. This study was aimed to assess the determinants of voluntary counseling and testing experience among students.

**Methods:**

An institution-based cross-sectional study was conducted between November and January, 2020. A simple random sampling procedure was used to select participants from the target group. The Chi-square test, descriptive analysis, and a binary logistic regression analysis were used to identify the factors associated with VCT experience among students.

**Results:**

Out of 398 participants, 42.5% experienced VCT services. From 59.5% of female participants in the study, only 22.9% experienced VCT services. The logistic regression results revealed that male participants less likely experienced VCT (AOR = 0.549; 95%CI: 0.330, 0.910; *p*=0.020) compared to female students. Students who had VCT service access in their surroundings (AOR = 2.348; 95%CI: 1.371, 4.020; *p*=0.002), a sexual partner (AOR = 3.795; 95%CI: 1.214, 11.868; *p*=0.022), and media access (AOR = 2.374; 95%CI: 1.059, 5.320; *p*=0.036) were more likely to utilize VCT services than their reference categories.

**Conclusions:**

VCT utilization among students remains limited. In this study, sex, age, region, the education of mother and father, having boy/girlfriend, the source of information, service access, media access, and attitude were the identified factors of VCT utilization. To enhance the usage of VCT services, the facilities should be publicized, and all information regarding VCT should be made available to teenagers.

## 1. Background

Voluntary counseling and testing is a confidential counseling process that allows a person to make an informed decision about learning his or her HIV status and obtaining recommendations on how to continue [1, 2]. On a global basis, it is well-recognized as a successful and necessary tool for HIV prevention and treatment [3]. According to 2020 WHO estimates, over 30% of all new HIV infections globally are estimated to occur among youths aged 15 to 25 years [4]. Almost 3.3 million youths in sub-Saharan Africa are HIV-positive [5]. It has a critical role in fostering behavioral change, reducing unprotected sex, and reducing the prevalence of HIV and other sexually transmitted infections [6]. HIV/AIDS is a major public health issue in many regions of the world. Finding HIV-positive people and enrolling them in care is critical to meeting the UNAIDS 90–90–90 targets of diagnosing 90% of HIV-positive people, initiating 90% of those diagnosed on ART, and attaining viral suppression in 90% of those on ART. To meet the first goal, no one should be denied access to HIV testing services [7].

At least 90% of people with HIV must be tested at least once, and this goal is being approached or set by several African countries [8, 9]. Voluntary counseling and testing (VCT) is one of the most important preventative techniques and a gateway to AIDS treatment. Ethiopia is one of the countries hardest hit by the HIV epidemic in sub-Saharan Africa, where unprotected heterosexual contact and mother-to-child transmission are the main modes of infection and VCT experience is extremely limited [1, 10–13].

In Ethiopia, one of the most important policy responses to the HIV/AIDS epidemic has been voluntary counseling and testing (VCT). However, the country's VCT experience has been limited [10]. According to several research findings in Ethiopia, volunteer counseling and testing service experience is limited, and its level of utilization varies across different groups of the community [14, 15]. It is also supported by studies conducted among university and college students. According to the research conducted in Bahir Dar, 38.6 percent of the university students were tested [16]. Another study in Debre Birhan found that 35.2 percent of college students were affected [17]. The epidemic continues to affect teenagers and adults, particularly adult and young women. Almost one-fourth of HIV patients are under the age of 25. According to the literature, young people aged 15 to 24 years are at the forefront of the HIV/AIDS epidemic, and VCT uptake is very low among university students, who are regarded to be a high-risk population for HIV/AIDS infection [5, 18, 19].

An essential preventive measure to lower HIV transmission is HIV testing. Uncertainty exists regarding the relationship between HIV-related knowledge and the use of HIV testing services [20]. Some achievements have been recorded after 40 years, however, significant obstacles are still there. Although the worldwide adoption of voluntary HIV counseling and testing (VCT) has increased, challenges still exist in some parts of the world because of a lack of funding, a lack of national priority, low levels of awareness and access to VCT programs, and overworked medical professionals [21, 22].

Youths in Ethiopia, particularly high school and university students, are among the most sexually active and HIV-positive young people. Yet, VCT uptake among this demographic is low, which is in the range of 35% to 38% [16]. Because of the tremendous influence of peer pressure and the formation of their sexual and social identities, youths are prone to HIV [23]. Preparatory school and university students have low knowledge, attitude, and practice (KAP) when it comes to VCT, according to many studies. Ignorance, fear of being positive, lack of a perception of being at risk, cost of VCT, insufficient number of VCT clinics, and stigmatization were identified to be key barriers to VCT for HIV acceptance [23–26].

There is a scarcity of research in Ethiopia addressing VCT acceptance or experience among university students, especially in these topic areas. Furthermore, teens account for a substantial proportion of HIV-positive people. As a result, the purpose of this study was to determine the prevalence of voluntary counseling and testing experiences among Mizan-Tepi University students and the characteristics that contributed to these experiences.

## 2. Methods and Material

### 2.1. Study Area

Mizan-Tepi University is a government university in Ethiopia that was founded in 2006GC. It includes three campuses (Mizan Campus in Mizan town, Tepi Campus in Tepi town, and Mizan-Aman Teaching Hospital), and this study was conducted on the Tepi campus. Tepi Campus is located in Sheka Zone, Tepi town, about 611 kilometers from Ethiopia's capital city. According to the Tepi campus's students and alumni management directorate, there are 16 departments within the College of Natural and Computational Sciences, College of Engineering Technology, and School of Computing and Informatics, with a total of 5,739 students, and 4,188 of them are male.

### 2.2. Study Design and Population

An institution-based cross-sectional study was conducted to assess the prevalence of the utilization of the voluntary counseling test (VCT) experience among university students between November and January, 2020. All Mizan-Tepi University Tepi campus students under two colleges and one school (College of Natural and Computational science, College of Engineering and Technology, and School of Computing and Informatics) were considered. All regular undergraduate students in the Tepi campus were selected as target population of this study. There is homogeneity among the study population, and the target population was known, therefore, the simple random sampling technique was used to select the sample from the target population by applying the lottery method.

### 2.3. Inclusion and Exclusion Criteria

All Mizan-Tepi university undergraduate regular students were illegible to be included in the study. Students attending nonregular class, being seriously ill, and not volunteering to participate in these study were excluded.

### 2.4. Sample Size Determination

The sample size was calculated using the single-population proportion formula using the following parameters: 95% confidence level (*Z*_*α*/2_=1.96), 5% margin of error (*d*=0.05), and by taking 37.8% prevalence of utilization of VCT from the previous study [16]. Then, the sample size (*n*) was determined as follows:

(1) $$\begin{array}{c} n=\frac{{\left(Z_{\alpha /2}\right)}^{2}p\left(1-p\right)}{d^{2}}=\frac{{\left(1.96\right)}^{2}0.378\left(1-0.378\right)}{0.0025}=\frac{3.8416*0.378*0.622}{0.0025}=362. \end{array}$$

By considering 10% of none response rate, the sample size was calculated as 398.

### 2.5. Data Collection Procedure and Instrument

After acquiring consent and voluntariness from each respondent, basic sociodemographic and socioeconomic data, as well as sexual-related information, were obtained using a pretested, semistructured, and self-administered questionnaire.

### 2.6. Study Variables

Voluntary counseling and testing (VCT) experience was used as a dependent variable and coded as 0 if the respondents did not experience VCT and 1 if the respondents experienced VCT at least once. The predictor variables included in this study were the sex of students, age of students, religion, region, mother's education, father's education, original residence of students, service access, source of information, having a sexual partner, media access, knowledge about VCT, and attitude towards VCT.

### 2.7. Statistical Data Analysis

The collected data were cleared, coded, and analyzed using SPSS version 24. The descriptive statistics, such as frequency and percentage, and inferential statistics, such as the chi-square and multiple logistic regression with adjusted odd ratio (AOR), were applied to identify the significant factors. The predictor variables that were significant in the univariable analysis at 25% (*p*-value <0.25) level of significance and the variables that were considered more important were included in the multiple logistic regression analysis. The estimated adjusted odds ratios (AOR) and 95% confidence intervals with a *p*-value less than 5% indicates that the variables were statistically significant in the multivariable analysis.

## 3. Results

### 3.1. Sociodemographic Characteristics of Study Participants

**Figure 1:**
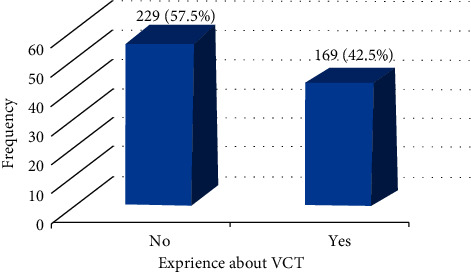
Prevalence of experience about VCT.

All 398 participants completed questionnaires with the response rate of 100%. About 57.5% of the participants had never experienced a voluntary counseling test, whereas the remaining 42.5% experienced one (Figure 1). Out of 398 participants included in this study, about 237(59.5%) was females, and of those females, only 91 (22.9%) experienced VCT services, while of the total male students included in the study, 83 (20.9%) had no experience about the VCT service. 192 participants (48.2%) in the age group of 21–22, 139 (35%) in the age group of 23 and above, 22 (5.5%) in the age group of 18–20, 106 (26.6%) in the age group of 21–22, and 41 (10.3%) in the age group of 23 and above experienced VCT services (Table 1).

Among the participants, 111 (27.9%) and 107 (26.9%) were from orthodox and protestant religion followers, respectively. About 60 (15.1%) from orthodox, 56 (14.1%) from protestant, 39 (9.8%) from Catholic, 51 (12.8%) from Muslim, and 23 (5.8%) from other religions did not experience VCT services. About 100 (25.1%) of the students were from the Oromia region, and of them, only 46 (11.6%) availed VCT services. From 65 (16.3%) participants from Addis Ababa, 24 (6.0%) experienced VCT services, and from 66 (16.6%) participants from SNNPR, only 25 (6.3%) experienced VCT services (Table 1).

According to the educational background, 135 (33.9%) and 91 (22.9%) of mothers and fathers of the students were uneducated, respectively, and students from those parents, i.e., only 60 (15.1%) and 27 (7%) experienced VCT services, respectively. However, 94 (23.6%) students were from mothers who attended primary school, and 74 (18.6%) were from fathers who attended primary school. Students with secondary and above educated parents were more experienced than those with uneducated parents. A majority of the students 269 (67.6%) reported having no voluntary counseling test (VCT) services in their surroundings. About 131 (32.9%) and 146 (36.7%) of students got information about voluntary counseling test (VCT) from their friends and health workers, respectively. Out of 398 participants, only 90 (22.6%) responded as they got information about VCT from different mass media. 253 (63.6%) students included in the study did not know about sexually transmitted infections (STI), and only 145 (36.4%) had information about sexually transmitted infections (STI) (Table 1).

This study revealed that most of the participants, i.e., 289 (72.6%), had no boyfriends or girlfriends, and of those participants, 156 (39.2%) did not experience VCT services, while 109 (27.4%) had boyfriends or girlfriends. 73 participants (18.3%) did not experience VCT services. 217 students (54.5%) included in this study were from rural areas, and of those participants, 90 (22.6%) did not experience VCT services. Out of 398 participants in the study, only 152 (38.2%) had media access, however, of those individuals, only 59 (14.8%) experienced VCT services. About 214 (53.8%) had negative attitudes toward VCT services. Among the participants, 218 (54.8%) had knowledge about the VCT services, while only 84 (21.1%) had practiced VCT (Table 1).

### 3.2. Association of Voluntary Counseling Test (VCT) of Students and Explanatory Variables

The results of the chi-square test of the association revealed that the sex of students, age of students, mother education level, father education level, service access, source of information, having a sexual partner, and attitude toward VCT had a statistically significant association with the voluntary counseling test (VCT) experience, whereas the remaining variables had no statistically significant relationship with the voluntary counseling test (VCT) experience (Table 1).

### 3.3. Factors Associated with Voluntary Counseling and Testing (VCT) Experience among Students

The results of binary logistic regression analysis revealed that sex, age, region, mother's education, father's education, service access, source of information, having a sexual partner, media access, and attitudes toward VCT service had statistically significant effects on the voluntary counseling and testing experience of students. Male students were less likely to have VCT experiences (AOR = 0.549; 95%CI: 0.330–0.910, *p*-value = 0.020) than female students. The odds ratio for the students whose age group was 21–22 (AOR = 2.896; 95%CI: 1.451–5.782, *p*-value = 0.003) implies that the students in the age group 21–22 were 2.896 times more likely to experience the VCT service than the reference category age group of 18–20. Students from Addis Ababa residence were more likely to practice VCT (AOR = 1.274; 95%CI: 1.139–2.541, *p*-value = 0.001) compared to students from Oromia region (Table 2).

The participants whose mothers attended the primary and secondary school were 3.544 and 1.413 times more likely to experience VCT (AOR = 3.544; 95%CI: 1.643–7.648, *p*-value = 0.001) and (AOR = 1.413; 95%CI: 1.185–3.919; *p*-value = 0.030) than students with uneducated mothers, respectively. Students from the fathers of different educational backgrounds had a statistically significant effect on VCT experiences. Respondents whose fathers had primary and above education had odds ratio greater than one, i.e., the odds ratio for the father who attended the primary class was (AOR = 3.332; 95%CI: 1.447–7.673, *p*-value = 0.005), secondary class was (AOR = 4.534; 95%CI: 1.953–10.524, *p*-value = 0.001), diploma was (AOR = 3.557; 95%CI: 1.394–9.071, *p*-value = 0.008), and degree and above was (AOR = 10.180, 95%CI: 3.843–26.965, *p*-value = 0.001). It implies that students with the fathers who were primary and above educated were more likely to experience VCT compared to students with uneducated father (Table 2).

Students with VCT services in their surroundings were more likely to experience VCT (AOR = 2.348; 95%CI: 1.371–4.020, *p*-value = 0.002) compared to students not having any VCT service access in their surroundings. Students whose information source was mass media had an odds ratio of (AOR = 3.221; 95%CI: 1.213–8.556, *p*-value = 0.019), which implies that students who got information about the VCT service from mass media were 3.221 times more likely to experience VCT services than students who got information from their parents.

The participants who had a boyfriend or girlfriend were 3.795 times more likely to practice VCT service compared to students who had no friends (AOR = 3.795; 95%CI: 1.214–11.868, *p*-value = 0.022). Media access plays a central role in sharing information in many directions. In this regard, the students who had media access in their surroundings had an odd ratio of (AOR = 2.374; 95%CI: 1.059–5.320, *p*-value = 0.036). This result shows that students who had media access in their surroundings were 2.374 times more likely to practice VCT services than students without media access. Respondents who had a positive attitude toward VCT services had an odds ratio of (AOR = 1.330; 95%CI: 1.173–2.838, *p*-value = 0.017), which implies that students with a positive attitude toward VCT services practice a voluntary counseling test 33% more than students with a negative attitude (Table 2).

## 4. Discussions

Voluntary counseling and testing (VCT) has been shown to be one of the most effective tools in the fight against HIV/AIDS. It is widely acknowledged as a critical component of HIV/AIDS preventive methods. Despite the fact that several studies have demonstrated the limited consumption of VCT services, particularly in developing countries, this study attempted to investigate the prevalence and associated determinants of VCT experience among Mizan-Tepi University students [27]. In this study, of all participants, only 42.5% experienced the voluntary counseling and testing services. This result is almost similar to the study conducted in the same area prior to this study [28, 29], higher than the prevalence of VCT utilization study conducted in the West Gojjam Merawi Preparatory school Ethiopia by 31.5% [1], Debre Birhan students training College by 35.19% [17], Bahir Dar University by 37.8% [16], and Addis Ababa University by 38.6% [30]. However, this result is lower than the prevalence of VCT utilization study conducted in the Debre Markos University by 58.5% [27] and the study done in the Northwestern parts among the university students by 61.8% [31]. It implies that the utilization of voluntary counseling and testing experience among university students were found to be very lower.

The funding of this study revealed that male participants were less likely to practice voluntary counseling and testing compared to female participants. This study is in line with the study conducted in the Addis Ababa university [32], Ambo University [33], in Ethiopia [34], in sub-Saharan Africa [35], in South Africa [36], in Lesotho [37], in Arusha City, Tanzania [38], and it contradicts with the study done in the West Gojjam Merawi Preparatory school [1]. It might be the awareness gap between male and female students in the high school level and also the increment of the female students confidence in the higher institution compared to the lower class.

Students in the age group of 21–22 were more likely to practice VCT than their counterparts in the age group 18–20. This result revealed that when participants age increases, the experience of using VCT among youths increases and is consistent with the study conducted in the Addis Ababa University and in Butajira Senior Secondary school [32, 39]. Students from Addis Ababa city administration practiced more compared to students from another regions and city administrations. The possible explanation may be enough information sources and service access in the city administration than other regions.

The experience for voluntary counseling and testing was higher for students who were from primary and above educated mothers and fathers compared to students from uneducated mothers and fathers. This finding is consistent with the study done in the Gondar town [40]. The possible explanation maybe that mothers and fathers who were educated at a level of primary and above increased awareness about voluntary counseling and testing advantages in their children. In other words, parents with different levels of education had the knowledge to share information about the service to their families compared to uneducated parents.

In this study, students who had access to a volunteer counseling and testing service in their area were 2.348 times more likely to be experienced than students who did not have access to a voluntary counseling and testing service. Volunteer counseling and testing services were provided by health professionals at health facilities, and hence, the lack of health facilities and professionals was the known reason for persons not participating in voluntary counseling and testing. This finding is in line with the study conducted in the Arusha City, Tanzania [38] and in Mwanza region, Tanzania [41].

Considering the source of information about voluntary counseling and testing service, mass media had the significant effect on VCT as the source of information compared to students who get information from their parents. This study is also similar to an earlier study done in southeast Nigeria among polytechnic students [42], Kenya [43], and Ethiopia [44], and it revealed that a majority of the students had knowledge of VCT with mass media. It means that students who got information from at least one of the listed sources (newspaper, television, radio, and internet) were considered students who got information from mass media.

Students who had sexual partners (boyfriend or girlfriend) were more likely to practice voluntary counseling and testing compared to students who did not. Those who had a boyfriend or girlfriend may have had a higher likelihood of having VCT as a precondition for starting sex, or they may have discussed VCT with their spouse, increasing their self-efficacy to have VCT. Those who have never had a boyfriend or girlfriend, on the other hand, may believe that they are not at risk since they have not yet had sex. This finding is in line with the study done in Bahirdar University [16], Harar college [45], and Tigray [46] in Kenya [47]. Being associated with someone increases the likelihood of using VCT services substantially more than not being involved. However, a study conducted among school students in Gurage zone [48] contradicts this study. It might be attributable to the research population (school adolescents) and the sample method (volunteer sampling), where singleness is the norm.

According to different studies in different parts of the country, an individual who had access to different media, such as newspaper, television, radio, and internet, were more likely to experience voluntary counseling and testing service compared to those students who had no media access. This study is in line with the study conducted in southeast Nigeria among students [42] in tertiary institutions in Enugu State Nigeria [3], Kilimanjaro region, and Tanzania, among healthcare professional students [24] in Ethiopia using EDHS data [49], and a systematic review of contingent valuation studies [50]. Students with a positive attitude toward volunteer counseling and testing service were more likely to engage in voluntary counseling and testing service than students with a negative attitude, according to this study. This study is consistent with the study conducted in Merawi Preparatory school students in West Gojjam [1].

## 5. Conclusion

According to this study and different studies conducted prior to this study, VCT utilization experience was limited in Ethiopia higher institutions until now. This study identified the factors that determine the VCT utilization of students in higher institutions, specifically, in Mizan-Tepi University, Tepi campus. The sex of the students, ages, region, mother's education level, father's education level, if they had boy/girlfriend, source of information, VCT service access, media access, and attitude towards VCT were identified as the significant factors for voluntary counseling and testing experience among students. To increase the use of VCT services, the VCT facilities should be publicly advertised, and all information about them must be made available to teenagers to raise awareness. Stand-alone VCT facilities should be built in communities and educational institutions to improve access to VCT services. Furthermore, community education programs in market, mass media, and other public places should be held to instruct parents who are accountable for their children.

## Figures and Tables

**Table 1:** Descriptive statistics and association test for voluntary counseling and testing experience status among MTU students.

Variables	Categories	Have you Experienced VCT?		Total (%)	Chi-Square	DF	*P*-value
		No	Yes				
		Count (%)	Count (%)				
Sex	Female	146 (36.7)	91(22.9)	237(59.5)	3.964	1	0.046
	Male	83 (20.9)	78 (19.6)	161 (40.5)			
Age	18–20	45 (11.5)	22 (5.5)	67 (16.8)	24.873	2	0.001
	21–22	86 (21.6)	106 (26.6)	192 (48.2)			
	23 and above	98 (24.6)	41 (10.3)	139 (35.0)			

Religion	Orthodox	60 (15.1)	51 (12.8)	111 (27.9)	6.763	4	0.149
	Protestant	56 (14.1)	51 (12.8)	107 (26.9)			
	Catholic	39 (9.8)	24 (6.0)	63 (15.8)			
	Muslim	51 (12.8)	36 (9.0)	87 (21.9)			
	Others	23 (5.8)	7 (1.8)	30 (7.5)			

Region	Oromia	54 (13.6)	46 (11.6)	100 (25.1)	5.396	6	0.494
	Tigray	17 (4.3)	9 (2.3)	26 (6.5)			
	Amhara	25 (6.3)	28 (7.0)	53 (13.3)			
	SNNPR	41 (10.3)	25 (6.3)	66 (16.6)			
	Addis Ababa	41 (10.3)	24 (6.0)	65 (16.3)			
	Gambella	13 (3.3)	12 (3.0)	25 (6.3)			
	Others	38 (9.5)	25 (6.3)	63 (15.8)			

Mother Education	Illiterate	75 (18.8)	60 (15.1)	135 (33.9)	9.244	4	0.045
	Primary School	63 (15.8)	31 (7.8)	94 (23.6)			
	Secondary School	42 (10.6)	24 (6.0)	66 (16.6)			
	Diploma	18 (4.5)	23 (5.8)	41 (10.3)			
	≥Degree	31 (7.8)	31 (7.8)	62 (15.6)			

Father Education	Illiterate	64 (16.1)	27 (6.8)	91 (22.9)	15.271	4	0.004
	Primary School	46 (11.6)	28 (7.0)	74 (18.6)			
	Secondary School	58 (14.6)	46 (11.6)	104 (26.1)			
	Diploma	35 (8.8)	29 (7.3)	64 (16.1)			
	≥Degree	26 (6.5)	39 (9.8)	65 (16.3)			

Service access	No	171 (43.0)	98 (24.6)	269 (67.6)	12.356	1	0.001
	Yes	58 (14.6)	71 (178)	129 (32.4)			

Source of information	Parent	17 (4.3)	14 (3.5)	31 (7.8)	25.589	3	0.001
	Friend	71 (17.8)	60 (15.1)	131 (32.9)			
	Health worker	69 (17.3)	77 (19.3)	146 (36.7)			
	Mass media	72 (18.1)	18 (4.5)	90 (22.6)			

Know about STI	No	138 (34.7)	115 (28.9)	253 (63.6)	2.545	1	0.111
	Yes	91 (22.9)	54 (13.6)	145 (36.4)			

Having sexual partner	No	156 (39.2)	133 (33.4)	289 (72.6)	5.469	1	0.019
	Yes	36 (9.0)	73 (18.3)	109 (27.4)			

Students residence	Urban	102 (25.6)	79 (19.8)	181 (45.5)	0.191	1	0.662
	Rural	127 (31.9)	90 (22.6)	217 (54.5)			

Media access	No	136 (34.2)	110 (27.6)	246 (61.8)	1.338	1	0.247
	Yes	93 (23.4)	59 (14.8)	152 (38.2)			

Attitudes toward VCT	Negative	111 (27.9)	103 (25.9)	214 (53.8)	6.088	1	0.014
	Positive	118 (29.6)	66 (16.6)	184 (46.2)			

Knowledge about VCT	No	95 (23.9)	85 (21.4)	180 (45.2)	3.047	1	0.081
	Yes	134 (33.7)	84 (21.1)	218 (54.8)			

**Table 2:** Factors associated with Voluntary counseling and testing experience among students.

Variables	Categories	*β*	AOR	95% CI for Exp (*β*)		*P*-value
				Lower	Upper	
Sex	Female	Ref.				
	Male	−600	0.549	0.330	0.910	0.020

Age	18–20	Ref.				
	21–22	1.063	2.896	1.451	5.782	0.003
	23 and above	−434	0.648	0.310	1.354	0.249

Region	Oromia	Ref.				
	Tigray	−389	0.678	0.221	2.077	0.496
	Amhara	0.042	1.042	0.463	2.346	0.920
	SNNPR	−308	0.735	0.343	1.572	0.427
	Addis Ababa	0.242	1.274	1.139	2.541	0.001
	Gambella	−429	0.651	0.193	2.193	0.488
	Others	−300	0.741	0.343	1.601	0.446

Mother Education	Illiterate	Ref.				
	Primary School	1.265	3.544	1.643	7.648	0.001
	Secondary School	0.346	1.413	1.185	3.919	0.030
	Diploma	0.293	1.340	0.476	3.769	0.579
	≥Degree	−229	0.796	0.351	1.806	0.585

Father Education	Illiterate	Ref.				
	Primary School	1.204	3.332	1.447	7.673	0.005
	Secondary School	1.512	4.534	1.953	10.524	0.001
	Diploma	1.269	3.557	1.394	9.071	0.008
	≥Degree	2.320	10.180	3.843	26.965	0.001

Service access	No	Ref.				
	Yes	0.854	2.348	1.371	4.020	0.002

Source of information	Parent	Ref.				
	Friend	0.644	1.904	0.736	4.927	0.184
	Health worker	−921	0.400	0.142	1.113	0.079
	Mass media	1.170	3.221	1.213	8.556	0.019

Having sexual partner	No	Ref.				
	Yes	1.334	3.795	1.214	11.868	0.022

Media access	No	Ref.				
	Yes	0.865	2.374	1.059	5.320	0.036

Attitudes toward VCT	Negative	Ref.				
	Positive	0.273	1.330	1.173	2.838	0.017

## Data Availability

The datasets used in this study are available from the corresponding author upon reasonable request.

## Abbreviations

VCT: Voluntary counseling and testing
AOR: Adjusted odds ratio
CI: Confidence interval
DF: Degree freedom
EDHS: Ethiopian demographic and health survey
HIV: Human immunodeficiency virus
AIDS: Acquired immunodeficiency syndrome.
